# Surgical management of benign noninfected urachal cysts in adult patients: two case reports

**DOI:** 10.1186/s13256-023-03944-8

**Published:** 2023-05-24

**Authors:** Asma Sghaier, Eya Lamloum, Mehdi Debaibi, Azza Sridi, Adnene Chouchene

**Affiliations:** Hospital of The Forces and The Security of Interior La Marsa Tunis, Faculty of Medicine of Tunis, University of Tunis El Manar, Tunis, Tunisia

**Keywords:** Urachal cyst, Management, Laparoscopy, Surgery, Case report

## Abstract

**Background:**

Abnormalities of the urachus include the patent urachus, cysts, sinus, and fistula. Each of these entities represents a failure of complete obliteration of the urachus. Contrary to other urachus anomalies, urachal cysts are usually small and silent unless they are infected. The diagnosis is often made during childhood. A benign noninfected urachal cyst discovered in adulthood is a rare condition.

**Case presentation:**

Herein we report two cases of benign noninfected urachal cysts in adults. The first case is a 26-year-old Tunisian white man who presented with complaints of clear fluid draining from the base of the umbilicus evolving for a week, with no other associated symptoms. The other case was 27-year-old Tunisian white woman who was referred to the surgery department with a history of intermittent draining of clear fluid from the umbilicus. The two cases had laparoscopic resection of urachus cysts.

**Discussion:**

Laparoscopy represents a good alternative for the management of persistent or infected urachus, especially when this is suspected, despite a lack of radiological evidence. Laparoscopy in the management of urachal cysts is safe, effective, and offers good cosmesis, with all the advantages of a minimally invasive approach.

**Conclusion:**

Managing persistent and symptomatic urachal anomalies requires a wide surgical excision. Such intervention is recommended to prevent symptom recurrence and complications, most notably malignant degeneration. A laparoscopic approach offers excellent outcomes, and is recommended to treat these abnormalities.

## Background

During the first stages of intrauterine life, the urachus connects the bladder to the allantois sac through the umbilicus. Toward the end of gestation, the urachus obliterates and becomes a fibrous band, forming the umbilical ligament between the peritoneum and fascia. Abnormalities of the urachus include the patent urachus, cysts, sinus, and fistula. Each of these entities represents a failure of complete obliteration of the urachus. Contrary to other urachus anomalies, urachal cysts are usually small and silent unless they are infected. The diagnosis is often made during childhood. Infection is the usual mode of presentation among adult cases, otherwise the condition usually remains asymptomatic. Benign noninfected urachal cysts discovered in adulthood is a rare condition considered.

## Case presentation

**Fig. 1:**
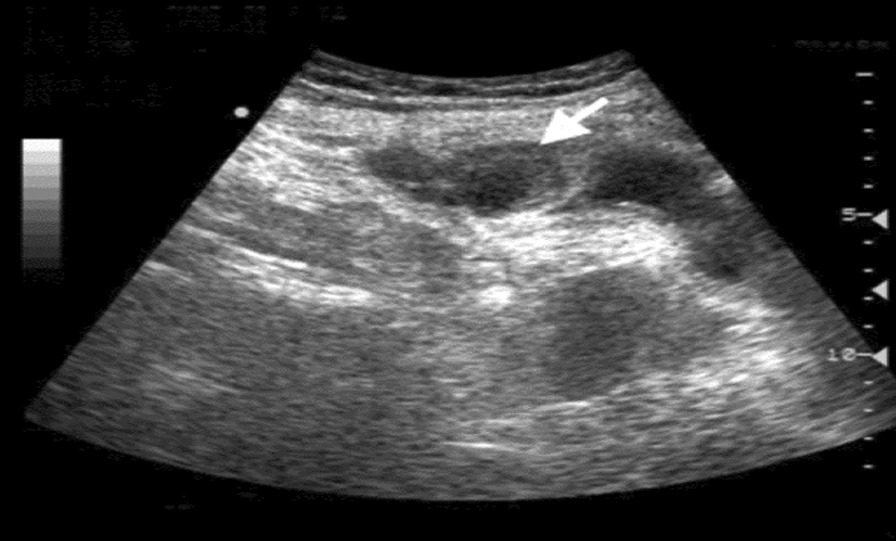
Abdominal ultrasonographic finding of a urachal cyst. Arrow indicating the urachal cyst

**Fig. 2:**
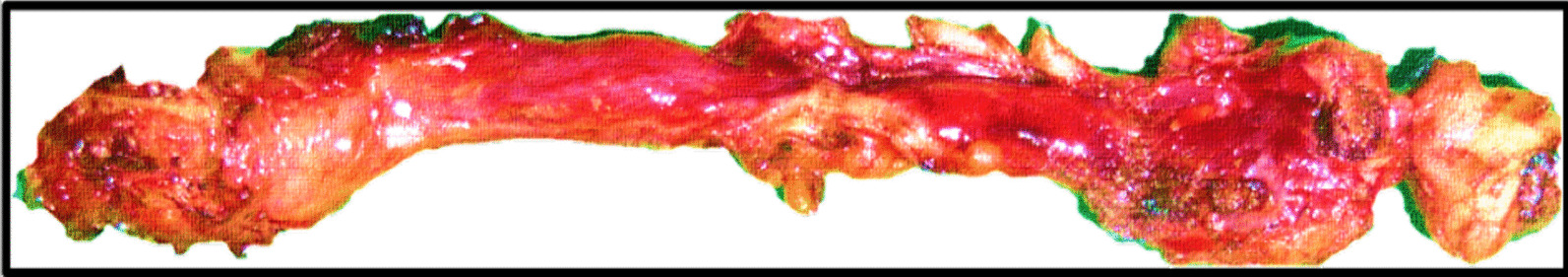
Urachal cyst after laparoscopic excision

We present the cases of two patients with benign noninfected urachal cysts manifested in adulthood. The first case is a 26-year-old Tunisian white man who presented with complaints of clear fluid draining from the base of the umbilicus evolving for a week, with no other associated symptoms. He had no prior history of a lower abdominal mass and no voiding complaints. The physical examination revealed an outcome of clear fluid from the umbilicus with no signs of inflammation or a palpable mass, associated with a noncomplicated umbilical hernia. The results of all blood tests were normal. A subsequent urine culture was negative. We completed abdominal sonography that showed a small peri vesical hypoechogenic cystic formation reminiscent of a urachal cyst (Fig. 1). The patient was then operated on: he had a laparoscopic excision of the cyst and was cured of an umbilical hernia in the same surgery (Fig. 2). The diagnosis of a benign noninfected urachal cyst was confirmed histologically. No adverse event was noted and the patient was discharged on day 1 after surgery.

The second case had similar clinical presentations. The patient was a 27-year-old Tunisian white woman who was referred to the surgery department with a history of intermittent draining of clear fluid from the umbilicus evolving for a year, with no other associated symptoms. The patient was treated with antibiotics, but recurrence of the symptomatology was noted. Blood and urine tests were normal. Abdominal sonography combined with a computed tomography (CT) scan identified a urachal cyst of 3 cm. the patient underwent surgery and had a laparoscopic excision of the urachal cyst with no intraoperative incidents. The postoperative course was simple. Pathologic examination revealed the urachal cysts without any unusual features.

For both patients, no recurrence of the symptoms was noted in the subsequent clinical follow-up.

## Discussion

The urachus is an embryologic structure that communicates between the apex of the bladder and the umbilicus. It normally closes by birth. If any portion of this embryologic structure remains patent, a urachal abnormality results. Various types of remnants have been described, including cysts, sinus, diverticulum, and a patent urachus. It is a rare congenital anomaly, with an incidence of 1:300,000 in infants and 1:5000 in adults [1].

Infection is the most common complication [1].

Urachal remnants, most commonly cysts, require intervention when they become infected or symptomatic.

As in the two reported cases, periumbilical drainage is the most common presentation of urachal cyst in adults and it is not necessarily associated with infection. Diagnosis is facilitated by imaging, especially ultrasound, which shows the cyst in the majority of cases. Otherwise, this examination can be replaced by a CT scan, especially in case of complications.

In Tables 1 and 2 we have summarized all the cases of benign noninfected urachal cyst in adult patients that have been reported in the literature so far.

**Table 1:** Case reports of benign noninfected urachal cysts in adult until December 2021

Year	Author	Country	Sex	Age	Initial symptoms	External drainage from the umbilicus	Explorations	Treatment	Final diagnosis
2021	Corsello *et al*. [1]	USA	M	39	Umbilical hernia, but he also noted drainage from the umbilicus	Yes	CT scan	Surgical excision of the cyst and cure of the umbilical hernia	Umbilical hernia with simple ruptured urachal cyst
2018	Singh *et al*. [2]	India	F	30	Lower abdominal pain with dysuria	No	CT scan, cystoscopy	Partial cystectomy with surgical resection of the cyst	Urachal cyst with xanthogranulomatous cystitis
2002	Milotic *et al*. [3]	Croatia	F	73	Dysuria	No	US, CT	Surgical resection, open laparotomy	Urachal cyst containing calculis
2008	Seo *et al*. [4]	Korea	M	58	Lower abdominal pain and urinary frequency	No	X-ray, US, CT	Surgical excision, laparoscopically	Urachal cyst containing large stones
2015	Okur *et al*. [5]	Turkey	F	17	Periumbilical pain	No	US, CT	Surgical excision mini laparotomy	Simple urachal cyst
2015	Kidger *et al*. [6]	UK	M	30		No	CT, cystoscopy	Cystectomy	Simple urachal cyst found incidentally after cystectomy
2010	Long and Lang [7]	USA	M	47		No	CT	Not specified	Simple noninfected urachal cyst
2001	Yagishita *et al*. [8]	Japan	M	79		No		Post mortem	Asymptomatic begnin noninfected, The cyst showed ovoid protrusion into urinary bladder cavity from the dome (3.5 × 2.0 × 2.0 cm in size)
2007	Castillo *et al*. [9]	Chili	M	38		No	CT	Surgical laparoscopic excision	Simple noninfected urachal cyst
2011	Zanghì *et al*. [10]	Italy	F	28	Dyspareunia	No	US, MRI	Laparoscopic surgical excision	Simple urachal cyst
2012	de Oliveira *et al*. [11]	Spain	M	24	Umbilical discharge	Yes	CT, cystoscopy	Laparoscopic surgical excision of the cyst	Urachal cyst
2016	Sakata *et al*. [12]	Australia	M	54	Mild lower abdominal pain with dysuria evolving for 1 month	No	CT, cystoscopy, colonoscopy	Surgical excision laparoscopically: primary anastomosis and en bloc resection of the urachal cyst and involved bladder	Peridiverticular abscess extending into a urachal cyst
2001	Yamada *et al*. [13]	Japan	F	48	Lower abdominal mass	No	US, MRI	Laparoscopic surgical excision	Simple urachal cyst
2005	Donate Moreno *et al*. [14]	Spain	F	32		No	US, MRI	Surgical resection	Incidental finding at pregnancy routine US
2005	Cherukupalli and Schein [15]	China	M	44					Simple urachal cyst
2011	Bella *et al*. [16]	Thailand	M	70	Lower abdominal mass	No	CT	Laparoscopic surgical resection	Simple urachal cyst
2011	Bella *et al*. [17]		F	32	Incidental finding on routine pregnancy US	No	US	Laparoscopic surgical resection	Simple urachal cyst

M = Male, F = female, US = ultra sonographic, MRI = magnetic resonance imaging, CT-scan = computed tomography scan

**Table 2:** Series of urachal remnants in adult patient and cases of benign noninfected urachal cysts

Year	Author	Country	Series	Urachal cysts
2019	Luo *et al*. [15]	China	7	1/7
2012	Yang *et al*. [16]	China	26	12/26
2011	Destri *et al*. [18]	Italy	13	6/13
2006	Madeb *et al*. [19]	USA	5	1/5

Asymptomatic, noninfected urachal cysts can be approached with watchful waiting, while infected urachal cysts almost require treatment that includes antibiotics, percutaneous drainage, or surgical removal. The therapeutic option depends on the presenting signs and symptoms, in addition to the individual operability and eventual surgical complications. There are two possible treatment modalities of this entity: either percutaneous drainage followed by surgical removal or one-stage open, laparoscopic, or robotic removal; the decision depends on the surgeon’s expertise and technologies involved in each surgical approach and the unique characteristics of the urachal cystic lesion being evaluated [20].

The nonoperative management of symptomatic urachal cyst is an acceptable approach and can be applied to infected urachal cysts after initial drainage. Infected cysts drained adequately may obliterate progressively and spontaneously. Ultrasonography is very useful for follow-up.

With regards to complications, Cutting *et al*, [21] reported peri-umbilical bleeding after surgery. Active umbilical bleeding was controlled laparoscopically in the repeat intervention. The blood supply at the umbilicus is from the branch artery of the inferior epigastric artery not from umbilical ligaments [22]. Wide circular resection of the fascia around the umbilicus should be avoided since it can risk injury to this branch. Omphalitis secondary to symptomatic urachal remnants often necessitates simultaneous resection of the umbilicus.

Furthermore, incomplete resection can lead to recurrence; therefore, appropriate debridement of the infected tissue is required [23, 24].

## Conclusion

Although it normally disappears to birth, part of the urachus may persist in few people. Urachal cysts can develop at any age. Urachal cysts are often not associated with any signs or symptoms; however, there are complications such as infection. In these cases, symptoms may include abdominal pain, fever, pain with urination, and/or hematuria. A laparoscopic approach is best to treat these abnormalities, with less comorbidity and undesirable events.

## Data Availability

Not applicable

## Declarations

### Registration of research studies

Not applicable. Not commissioned, externally peer-reviewed.

## References

[1] Corsello J, Morris M, Denning D, Munie S (2021). Case of infected urachal cyst in an adult presenting as an incarcerated umbilical hernia. Am Surg.

[2] Singh A, Prasad HK, Shetty KJ, Philip NR, Salma R, Chakravarthy A (2018). Urachal cyst with xanthogranulomatous cystitis: a rare case report. Urol Ann.

[3] Milotic F, Fuckar Z, Gazdik M, Cicvaric T, Milotic I, Zauhar G (2002). Inflamed urachal cyst containing calculi in an adult. J Clin Ultrasound.

[4] Seo IY, Han DY, Oh SJ, Rim JS (2008). Laparoscopic excision of a urachal cyst containing large stones in an adult. Yonsei Med J.

[5] Okur SK, Pülat H, Karaköse O, Zihni I, Özçelik KÇ, Eroğlu HE (2015). A urachal cyst case with painful mass locates at ileal mesentery. Case Rep Surg.

[6] Kidger E, Stahlschmidt J, Garthwaite M, Fulford S, Southgate J, Baker SC (2016). A rare urachal cyst in a case of ketamine-induced cystitis provides mechanistic insights. Urology.

[7] Long SS, Lang E (2010). Urachal sinus cyst. J Urol.

[8] Yagishita H, Nagayama T, Zean Z, Ihara F, Hatori T, Nonaka H, Akima M (2001). A case of asymptomatic urachal cyst in autopsy—histopathological study of urachal cyst and review of the literature of 99 cases during a 10 year period in Japan. Hinyokika kiyo.

[9] Castillo OA, Vitagliano G, Olivares R, Sanchez-Salas R (2007). Complete excision of urachal cyst by laparoscopic means: a new approach to an uncommon disorder. Arch Esp Urol.

[10] Zanghì A, Cavallaro A, Di Vita M, Piccolo G, Barbera G, Di Mattia P, Cappellani A (2011). An unique case of dyspareunia leading to the diagnosis of urachal cyst in a nulliparous 28-year-old woman. Clin Ter.

[11] de Oliveira MC, Vila F, Versos R, Araújo D, Fraga A (2012). Laparoscopic treatment of urachal remnants. Actas Urol Esp (Engl Ed).

[12] Sakata S, Grundy J, Naidu S, Gillespie C (2016). Urachal-sigmoid fistula managed by laparoscopic assisted high anterior resection, primary anastomosis and en bloc resection of the urachal cyst and involved bladder. Asian J Endosc Surg.

[13] Yamada T, Okamoto Y, Kasamatsu H, Mori H (2001). Laparoscopic-assisted removal of a large urachal cyst. J Am Assoc Gynecol Laparosc.

[14] Donate Moreno MJ, Giménez Bachs JM, Salinas Sánchez AS, Lorenzo Romero JG, Millán H, Pastor Guzmán JM (2005). Urachal pathology: an overview review and report of three clinical cases. Actas Urol Esp.

[15] Luo X, Lin J, Du L, Wu R, Li Z (2019). Ultrasound findings of urachal anomalies. A series of interesting cases. Med Ultrasonogr.

[16] Yang H, Zhou S, Tan C, Zhang B, Situ W (2012). Multislice spiral computer tomography imaging characteristics of urachus lesions. Zhong Nan Da Xue Xue Bao Yi Xue Ban.

[17] Permpongkosol S, Bella AJ, Suntisevee S, Leenanupunth C, Stoller ML (2011). Laparoscopic excision of urachal cysts in elderly men and woman following pregnancy. J Med Assoc Thai.

[18] Destri GL, Schillaci D, Latino R, Castaing M, Scilletta B, Cataldo AD (2011). The urachal pathology with umbilical manifestation: overview of laparoscopic technique. J Laparoendosc Adv Surg Tech.

[19] Madeb R, Knopf JK, Nicholson C, Donahue LA, Adcock B, Dever D, Tan BJ, Valvo JR, Eichel L (2006). The use of robotically assisted surgery for treating urachal anomalies. BJU Int.

[20] Lipskar AM, Glick RD, Rosen NG, Layliev J, Hong AR, Dolgin SE, Soffer SZ (2010). Nonoperative management of symptomatic urachal anomalies. J Pediatr Surg.

[21] Cutting CW, Hindley RG, Poulsen J (2005). Laparoscopic management of complicated urachal remnants. BJU Int.

[22] Stokes RB, Whetzel TP, Sommerhaug E, Saunders CJ (1998). Arterial vascular anatomy of the umbilicus. Plast Reconstr Surg.

[23] Sasaki H, Kimura S, Shimada H, Murakami M, Yanagisawa T, Atsuta M, Matsuura T, Yokawa Y, Ishida K, Egawa S (2018). Outcomes of laparoscopic resection of urachal remnants followed by novel umbilicoplasty. Int Urol Nephrol.

[24] Cherukupalli C, Schein M (2005). Urachal cyst causing intestinal obstruction. Int Surg.

